# Manic episode following lurasidone initiation in bipolar I disorder: a case report

**DOI:** 10.3389/fpsyt.2026.1853542

**Published:** 2026-06-09

**Authors:** Nasser M. Alzain, Feras A. Al-Awad, Saleh A. Alzahrani, Fahad M. Almsned

**Affiliations:** 1 Department of Psychiatry, College of Medicine, Imam Abdulrahman Bin Faisal University, Dammam, Saudi Arabia; 2 Research Center, King Fahad Specialist Hospital in Dammam (KFSH-D), Dammam, Saudi Arabia; 3 Research Program, Academic, Training, and Research Administration, Eastern Health Cluster, Dammam, Saudi Arabia; 4 School of Systems Biology, George Mason University, Fairfax, VA, United States

**Keywords:** antipsychotics, bipolar disorder, case report, lurasidone, mood stabilizers

## Abstract

Lurasidone is a second-generation antipsychotic approved for the treatment of bipolar depression. Although effective, antipsychotics may precipitate manic episodes in susceptible individuals. This report aims to describe a unique case of a patient with bipolar I disorder who developed acute mania following lurasidone titration, highlighting the need for vigilance during treatment initiation. We present the case of a 43-year-old man with a history of bipolar I disorder who presented with a severe depressive episode, characterized by depressed mood, insomnia, anhedonia, and suicidal ideation. Laboratory investigations revealed a subtherapeutic serum valproate level of less than 13 µg/mL (reference range: 50–100 µg/mL). Lurasidone therapy was initiated and gradually titrated to 120 mg daily. After 1 week, the patient exhibited increased energy and decreased sleep needs. By the second week, he developed a full manic episode, presenting an elevated mood, irritability, intrusive behavior, and sleeping only one to two hours per night. Following the immediate discontinuation of Lurasidone, alongside the optimization of his sodium valproate dosage and the reinitiation of paliperidone, the patient experienced a dramatic reduction in his manic symptoms. His clinical condition stabilized, and he was subsequently discharged. This case emphasizes the critical importance of close monitoring for manic symptoms following lurasidone initiation in patients with bipolar I disorder and inadequate mood stabilization.

## Introduction

Second-generation antipsychotics play a central role in the treatment of bipolar depression and psychosis. We report a case of a manic episode that occurred in a patient with bipolar I disorder who was undergoing lurasidone titration for the treatment of bipolar depression. Clinical guidelines currently recommend agents such as Lurasidone and quetiapine as first-line options for bipolar I depression due to their demonstrated antidepressant efficacy and acceptable tolerability profiles (1, 2). Randomized controlled trials have shown significant improvement in depressive symptoms with lurasidone monotherapy compared to placebo (2).

Despite their established antidepressant efficacy, second-generation antipsychotics have been associated with a phenomenon known as a “manic switch,” a rapid transition from bipolar depression into mania (3). Reviews of the literature have identified multiple cases of atypical antipsychotic associated mania or hypomania with specific case reports and case series documenting this adverse reaction during lurasidone treatment (4, 5). The proposed mechanism for this manic switch involves the serotonergic modulation of dopamine pathways (6). Lurasidone acts as a dopamine D2 receptor antagonist and a serotonin 5-HT2A and 5-HT7 receptor antagonist, while also exhibiting partial agonist activity at 5-HT1A receptors (7). This unique receptor profile increases dopamine and norepinephrine release in the prefrontal cortex, which regulates mood and cognition (8). While the partial agonism at 5-HT1A receptors and antagonism at 5-HT7 receptors effectively improve depressive symptoms, it may simultaneously increase dopamine release in cortical and limbic regions, thereby precipitating mania in susceptible patients.

Although Lurasidone is generally well tolerated, close monitoring of patients is highly recommended after initiating or increasing the dosage, particularly for those with bipolar I disorder and a history of prior manic episodes. Early identification of manic symptoms allows for prompt intervention and significantly reduces morbidity. We describe the clinical characteristics of lurasidone-induced mania to draw clinicians’ attention to the risks of titrating antipsychotics without adequate mood stabilizer coverage.

## Case report

A 43-year-old male presented to the emergency department requesting a medication refill and psychiatric follow-up. He had a known history of bipolar I disorder, initially diagnosed in 2001, with four prior psychiatric admissions; the most recent being in 2022 for a manic episode. He had no family history of mental illness or substance misuse. His prior pharmacological regimen included sodium valproate and risperidone, though he reported inconsistent adherence to the mood stabilizer. He denied any history of substance abuse or co-existing medical illnesses.

The patient’s baseline functioning had remained stable until September 2025, following the passing of his grandmother. Following this bereavement, he developed a persistent depressed mood, anhedonia, impaired concentration, and profound feelings of guilt. He avoided family gatherings and was unable to attend the funeral. His sleep was significantly reduced to approximately 3 hours per night, with early-morning awakenings. He also reported recurrent thoughts of death, such as drowning or ingesting cleaning powder, though without a specific plan or preparatory behavior. He thoughtfully identified his religious beliefs, attachment to his mother, and responsibility toward his children (who lived with his ex-wife) as protective factors against self-harm.

Over the subsequent months, his overall functioning progressively declined. He withdrew socially, experienced interpersonal conflicts with family members, missed a week of work, and began neglecting his personal hygiene. Additional psychosocial stressors, including financial difficulties and a recent motor vehicle accident, further exacerbated these depressive symptoms.

Upon mental state examination, the patient exhibited reduced grooming and poor personal hygiene. He remained cooperative with normal psychomotor activity. His speech was spontaneous but notably reduced in rate and volume. He subjectively described his mood as “not happy,” and his affect was observed to be depressed and congruent with his mood. While his thought form remained organized, he expressed persecutory and referential beliefs. He reiterated his suicidal ideation without a concrete plan and showed no evidence of hallucinations. The patient remained alert and fully oriented with intact cognition, though his insight and judgment were deemed limited. The choice of Lurasidone was not a simple default but the result of a careful, constrained clinical reasoning process that excluded all usual alternatives based on evidence.

Comprehensive laboratory investigations revealed normal hematologic, renal, hepatic, and thyroid function, along with unremarkable electrolyte parameters. The comprehensive toxicology screening was negative. The serum valproate level was below 13 µg/mL, which is significantly lower than the therapeutic range of 50 to 100 µg/mL, confirming the suboptimal mood stabilizer state of this patient at the time of presentation.

The patient was voluntarily admitted by the on-call clinician with a diagnosis of bipolar I disorder, currently in a severe depressive episode without psychosis. His prior paliperidone palmitate long-acting injection was discontinued upon admission. Alternative pharmacological interventions were systematically excluded; lamotrigine was avoided due to a prior history of lamotrigine-induced mania, quetiapine was contraindicated due to a previous intolerance related to profound sedation, and lithium was deemed inappropriate given his established history of poor adherence. The clinical course is summarized in **Table 1**.

**Table 1:** Clinical timeline of patient presentation, intervention, and outcomes.

Time point	Clinical event	Medication/intervention	Findings/notes
2001 – Pre-September 2025	Initial Diagnosis & Stable Baseline	Sodium valproate 2,000 mg nightly Risperidone 4 mg nightly	Bipolar I disorder diagnosed in 2001; four prior psychiatric admissions (most recent: 2022, manic episode); inconsistent adherence to mood stabilizer
September 2025	Depressive Episode Onset	No clinical contact; regimen unchanged	Triggered by bereavement (grandmother’s passing); depressed mood, anhedonia, impaired concentration, guilt, social withdrawal, sleep ~3 hrs/night, passive suicidal ideation (no plan or intent)
October – November 2025	Progressive Functional Decline	No clinical contact; regimen unchanged	Social withdrawal, interpersonal conflicts, missed work, self-neglect; compounding stressors: financial difficulties, motor vehicle accident
3 November 2025 (Admission — Day 0)	Emergency Presentation & Voluntary Psychiatric Admission	Paliperidone palmitate LAI: discontinued Lurasidone: initiated at 40 mg/day	Diagnosis: Bipolar I disorder — severe depressive episode without psychosis MSE: depressed affect, persecutory/referential beliefs, suicidal ideation, limited insight Valproate: <13 µg/mL (therapeutic range: 50–100 µg/mL) Excluded alternatives: lamotrigine (prior drug-induced mania), quetiapine (sedation intolerance), lithium (poor adherence)
9 November 2025 (Day 6 — Titration to 80 mg)	Early Hypomanic Signals Emerge	Lurasidone uptitrated to 80 mg/day (titration continued despite signals)	Decreased need for sleep, increased irritability, subjective increased energy (‘feeling energetic’); emerging hypomanic signals not treated as a clinical stop signal
Days 7–10 (Titration to 120 mg)	Target Dose Reached; Hypomanic Features Persist	Lurasidone uptitrated to 120 mg/day	Therapeutic target dose reached; hypomanic symptoms not formally reassessed prior to completing titration
13 November 2025 (Day 10 Post-Initiation)	Full Manic Episode Developed	Lorazepam 2 mg TID added (proved entirely ineffective)	Profound irritability, grandiose delusions (‘flying to the sky’), elevated mood, highly intrusive behaviour, sleep reduced to 1.5 hrs/night; diagnostic criteria for acute mania met
16 November 2025 (Manic Switch Recognition)	Lurasidone Discontinued; Antimanic Regimen Initiated	Lurasidone: DISCONTINUED Sodium valproate: optimised to 3,000 mg/night Oral paliperidone: 6 mg/day initiated	Dramatic reduction in manic symptoms upon discontinuation Naranjo ADR Probability Score = 9 → ‘Definite’ adverse drug reaction (score ≥9)
6 January 2026 (Discharge)	Clinical Stabilisation; Discharge in Stable Condition	Sodium valproate 3,000 mg nightly Paliperidone palmitate 150 mg IM monthly	Extended inpatient stay (~64 days) for full mood stabilisation; discharged on long-acting injectable antipsychotic to mitigate adherence risk Patient reported marked subjective improvement and expressed willingness to maintain prescribed regimen

*ADR, adverse drug reaction; ED, emergency department; IM, intramuscular; LAI, long-acting injectable; MSE, mental state examination; TID, three times daily.

Lurasidone therapy was subsequently initiated at a dose of 40 mg once daily and gradually titrated until reaching a dose of 120 mg daily. Lurasidone was started at 40 mg once daily and up-titrated to 120 mg once daily based on evidence of its efficacy in bipolar depression related to dopamine and serotonin receptor modulation (2, 9). However, when the titration reached a dose of 80 mg once daily, the patient began to exhibit early signs of hypomania, including a decreased need for sleep, increased irritability, and subjective feelings of being “energetic.” Despite these emerging symptoms, the titration continued until the 120 mg once-daily target was reached.

Approximately two weeks after the initiation of Lurasidone, the patient developed a full-blown manic episode. He became unmanageable, exhibiting profound irritability, engaging in significant interpersonal conflicts, and displaying an excessively elevated mood. His sleep was further reduced to merely 1.5 hours per night, accompanied by increased energy, frequent showering, and highly intrusive social behavior. He vividly described his mood as “flying to the sky” and presented it with grandiose delusions. These symptoms met criteria for an acute manic episode. To manage the agitation, lorazepam was administered at a dose of 2 mg three times per day as soon as the early signs appeared, but this intervention proved entirely ineffective.

The Lurasidone was discontinued immediately upon the recognition of the lurasidone-induced manic switch, resulting in a dramatic improvement in manic symptoms. Concurrently, his sodium valproate dosage was optimized to 3000 mg to reestablish therapeutic levels, given its established efficacy in acute mania and maintenance treatment (1, 10). Oral paliperidone at a dose of 6 mg daily was also initiated to control the persistent psychotic and manic symptoms. After removing the offending agent and reintroducing robust mood stabilizers and antipsychotic treatment, the patient’s clinical status improved, and he was discharged in a stable condition on sodium valproate 3000 mg nightly and paliperidone palmitate 150 mg intramuscular monthly injections. Following the discontinuation of lurasidone and the optimization of his mood stabilizer regimen, the patient reported a marked subjective improvement in his overall well-being. He acknowledged the episode retrospectively and expressed willingness to maintain adherence to his prescribed discharge regimen.

## Discussion

This case report demonstrates the temporal relationship between lurasidone titration and a manic switch during bipolar depression treatment. The temporal sequence is strongly suggestive: Initially, the patient presented with classic depressive symptoms, including depressed mood, anhedonia, suicidal ideation, and reduced sleep with no antecedent manic features. Crucially, his serum valproate concentration remained subtherapeutic upon admission, a critical factor that heightened his vulnerability to relapse. This case is unique in that it reports the unusual confluence of three independent vulnerability factors present at the time of lurasidone initiation: an extremely low serum valproate concentration of less than 13 µg/mL, active and compounding psychosocial stressors of bereavement, financial hardship, and a recent motor vehicle accident, and a well-established behavioral pattern of poor medication adherence. The simultaneous presence of these pharmacological, psychological, and behavioral risk factors in a single clinical encounter constitutes an unusually high-risk prescribing scenario that is rarely captured in its entirety in a single published case report. To formally characterize the causal relationship between Lurasidone and the manic episode, the Naranjo Adverse Drug Reaction Probability Scale was applied. In this case, scoring was based on the following: the existence of previous conclusive published reports of lurasidone-induced mania (+1); the clear temporal emergence of manic symptoms following lurasidone initiation (+2); dramatic symptom resolution upon discontinuation (+1); the absence of any plausible alternative cause (+2); dose-dependent worsening of symptoms as titration progressed from 80 mg to 120 mg (+1); a prior history of lamotrigine-induced mania suggesting a personal vulnerability to drug-induced mood switches (+1); and objective clinical documentation of deterioration and recovery (+1), which yielded a total score of 9, placing this case in the “Definite” adverse drug reaction category (score ≥9). This category provides the most robust level of standardized causal attribution, imparting structured, objective clinical narrative credibility beyond mere temporal association.

The onset of the manic episode followed the gradual titration of Lurasidone to 120 mg daily. The timeline of this emergence, spanning days to weeks, aligns closely with prior case reports detailing manic switches after initiating or increasing Lurasidone. The patient exhibited cardinal manic symptoms, including elevated mood, grandiosity, extreme energy, and a profound decrease in the need for sleep. Furthermore, the rapid and dramatic resolution of these symptoms following the discontinuation of Lurasidone and the re-optimization of his antimanic regimen solidifies the diagnosis of a lurasidone-induced manic episode.

This case further provides a rare and didactically instructive illustration of a critical decision point in dose escalation. When Lurasidone reached 80 mg daily, the patient began exhibiting early hypomanic features — decreased need for sleep, heightened irritability, and subjective feelings of increased energy. Despite these emerging signals, titration continued to the target dose of 120 mg daily. This highlights a major clinical judgment confound: The continuation of titration in the face of emerging hypomanic signals may reflect prescriber decision-making under uncertainty — a scenario in which the treating clinician may have interpreted these early features as transient side effects rather than prodromal indicators of a manic switch, or may have prioritized the imperative to reach the minimally effective therapeutic dose. This uncertainty does not preclude Lurasidone as the causative agent for this manic episode but highlights the inherent difficulty of real-world pharmacological titration in bipolar patients, where the separation between activation adverse events and therapeutic response may only be identified retrospectively. The dose escalation/therapeutic target dilemma versus early warning signals of mood destabilization is rarely explicitly described in case reports; however, it is immediately and very pragmatically relevant to prescribing. The message is clear: early signs of hypomania should be a clinical stop signal rather than a challenge in the titration of a drug.

From a pharmacological perspective, this phenomenon is well described in the literature. Lurasidone effectively blocks 5-HT7 receptors while partially stimulating 5-HT1A receptors, a receptor profile that significantly increases dopamine release in the prefrontal cortex and limbic regions (8). While this enhanced dopaminergic transmission is the precise mechanism by which Lurasidone exerts its potent antidepressant effects, an “overshoot” in this system can precipitate mania in susceptible individuals. Antipsychotic-induced mania, driven by these complex serotonergic modulations of dopamine pathways, has been documented across several second-generation antipsychotics (4). Furthermore, unlike most published case reports, where Lurasidone is one among several options, this case is clinically realistic: lamotrigine, quetiapine, and lithium were all contraindicated in this patient. Therefore, this case adds clinical nuance and direct translational applicability to those reported in the literature.

This case also underscores the critical importance of maintaining adequate mood stabilizer coverage during the treatment of bipolar depression. Therapeutic mood stabilizer levels are essential in mitigating the risk of a manic switch when initiating antidepressant therapies (1). In this instance, the patient’s persistently subtherapeutic valproate level significantly compromised his mood stability, rendering him exceptionally vulnerable to the activating effects of lurasidone titration. Notably, unlike the majority of published case reports documenting antipsychotic-induced manic switches, this case provides a precisely quantified serum mood stabilizer concentration at the time of the adverse event. With a valproate level below 13 µg/mL, less than a quarter of the lower limit of the therapeutic range (50–100 µg/mL), this provides pharmacokinetic specificity in support of the vulnerability hypothesis, and thus, a degree of scientific rigor that narrative case descriptions cannot achieve. Close clinical observation for early signs of hypomania, such as decreased need for sleep, increased energy, irritability, and behavioral changes, is crucial when starting or increasing lurasidone dosages. Early detection allows for the prompt discontinuation of the offending agent and the rapid initiation of appropriate antimanic treatments.

## Conclusion

This case highlights the substantial risk of a manic switch associated with lurasidone therapy during the treatment of bipolar depression in patients with bipolar I disorder. Clinicians must maintain a high index of suspicion and closely monitor patients following lurasidone initiation or dose escalation, particularly when mood stabilizer levels are known or suspected to be subtherapeutic. Early recognition of emergent manic symptoms and prompt treatment adjustment are vital strategies to improve clinical stability, ensure patient safety, and minimize the risk of subsequent relapse.

Three clinically actionable lessons emerge from this case. First, early hypomanic signals must be treated as a clinical stop signal, not a titration challenge. The emergence of decreased need for sleep and irritability at 80 mg should have prompted reassessment rather than continuation of dose escalation. A structured monitoring protocol — incorporating weekly mood charting, sleep logs, and collateral informant reporting — should accompany lurasidone titration schedules in all high-risk patients. Second, constrained prescribing contexts must be recognized as a category of heightened pharmacological risk. Patients in whom multiple first-line agents are contraindicated or not tolerated — as in this case — represent a clinically vulnerable subgroup requiring heightened vigilance when any activating pharmacological agent is introduced, as the margin for therapeutic maneuver is significantly narrowed. Third, rapid discontinuation remains the cornerstone of management. Consistent with other published reports, prompt discontinuation of Lurasidone in this case led to dramatic and sustained symptom resolution, reinforcing the need for decisive and timely clinical action whenever a drug-induced manic switch is suspected.

## Data Availability

The original contributions presented in the study are included in the article/supplementary material. Further inquiries can be directed to the corresponding author.
